# Plasma Biomarkers Associated With Heart Failure Hospitalization Among Patients With Atrial Fibrillation and Subtypes of Heart Failure

**DOI:** 10.1161/JAHA.125.045970

**Published:** 2026-01-14

**Authors:** Tymon Pol, Johan Lindbäck, Jonas Oldgren, John H. Alexander, Agneta Siegbahn, Lars Wallentin, Ziad Hijazi

**Affiliations:** ^1^ Department of Medical Sciences, Cardiology Uppsala University Uppsala Sweden; ^2^ Uppsala Clinical Research Center Uppsala Sweden; ^3^ Duke Clinical Research Institute Durham NC; ^4^ Department of Clinical Chemistry Uppsala University Uppsala Sweden

**Keywords:** atrial fibrillation, biomarkers, heart failure, Atrial Fibrillation, Heart Failure

## Abstract

**Background:**

Atrial fibrillation is associated with heart failure (HF) through a complex cause‐and‐effect relationship. We performed multiplex screening of plasma proteins in patients with atrial fibrillation to identify biomarkers and pathways associated with hospitalization for HF. Additionally, we aimed to identify potential pathophysiological differences between HF with reduced ejection fraction and HF with preserved ejection fraction at baseline in patients with atrial fibrillation.

**Methods:**

Using a case–cohort design of patients with atrial fibrillation from the ARISTOTLE (Apixaban for Reduction in Stroke and Other Thromboembolic Events in Atrial Fibrillation) trial, 596 cases with HF hospitalizations during follow‐up and 4029 randomly selected controls without HF hospitalization. Plasma obtained at randomization was analyzed with conventional immunoassays and proximity extension assay panels. Biomarker associations with HF hospitalization were evaluated using random survival forest, Boruta, and Cox‐regression analyses. Associations between biomarkers and HF subtype were evaluated with Wilcoxon−Mann–Whitney test with Bonferroni–Holm adjustment for multiplicity.

**Results:**

The biomarkers most strongly and significantly associated with increased risk of HF hospitalization after adjustment for clinical characteristics, renal function, and cardiac biomarkers, and after correction for multiplicity (*P*≤0.00027), were NT‐proBNP (N‐terminal pro‐B‐type natriuretic peptide), BNP (B‐type natriuretic peptide), hs‐cTnT (high‐sensitivity cardiac troponin T), fibroblast growth factor 23, spondin 1, insulin‐like growth factor binding protein 7, urokinase‐type plasminogen activator receptor, osteopontin, pentraxin‐related protein 3, and transferrin receptor protein 1R. Among patients with prevalent HF, 9 biomarkers remained significant after adjustment for multiplicity; NT‐proBNP, BNP, hs‐cTnT, renin, angiotensin‐converting enzyme 2, growth differentiation factor 15, and interleukin‐6 levels were higher in HF with reduced ejection fraction, whereas levels of stem cell factor and leptin were higher in HF with preserved ejection fraction (all *P*<0.05).

**Conclusions:**

Of 268 evaluated biomarkers, this study identified biomarkers representing mechanisms strongly associated with subsequent HF hospitalization. HF with reduced ejection fraction was more strongly associated with cardiorenal dysfunction and inflammation markers, while HF with preserved ejection fraction was associated with adipose metabolism and tissue repair proteins.

**Nonstandard Abbreviations and Acronyms**
ARISTOTLE: Apixaban for Reduction in Stroke and Other Thromboembolic Events in Atrial Fibrillation
FGF‐23: fibroblast growth factor 23
GDF‐15: growth differentiation factor 15
HFrEF: heart failure with reduced ejection fraction
HFpEF: heart failure with preserved ejection fraction
HHF: hospitalization for heart failure
IGFBP‐7: insulin‐like growth factor binding protein 7
PEA: proximity extension assay
PTX3: pentraxin‐related protein
SCF: stem cell factor
TR: transferrin receptor protein 1

**Clinical Perspective**

**What Is New?**

- In this multiplex proteomic study of patients with atrial fibrillation, biomarkers most strongly associated with heart failure hospitalization reflected cardiorenal dysfunction, growth factors, fibrinolysis, inflammation, and iron metabolism.
- Among patients with heart failure at baseline, those with heart failure with reduced ejection fraction exhibited higher levels of markers related to cardiorenal dysfunction, renin–angiotensin–aldosterone system activation, and oxidative stress/inflammation, whereas those with heart failure with preserved ejection fraction showed higher levels of adipose tissue metabolism, vasculogenesis, and tissue repair proteins.

**What Are the Clinical Implications?**

- The identified biomarker signatures deepen mechanistic insight, may enhance phenotyping, improve risk stratification, and inform future research toward targeted therapeutic strategies for this patient population.

Atrial fibrillation (AF) is associated with a 5‐fold increase in the risk of heart failure (HF), and these conditions frequently coexist.^1^ The cause‐and‐effect relationship between AF and HF is complex, and the precise pathophysiological mechanisms leading to HF in patients with AF remain unclear. HF can be classified according to the left ventricular ejection fraction (LVEF) into heart failure with preserved ejection fraction (HFpEF) and heart failure with reduced ejection fraction (HFrEF).^2^ While HFpEF and HFrEF have similar risk factors and clinical presentation, their disease pathogenesis is believed to be distinct and is poorly understood.^2^, ^3^ Therefore, there is a need for a deeper understanding of their underlying mechanisms.

Circulating biomarkers may offer valuable insights into cardiovascular as well as noncardiovascular mechanisms, and their measurements can aid in identifying patients at risk, shed light on key pathophysiologic processes involved in HF development, and may highlight potential treatment targets. Although BNP (B‐type natriuretic peptide) and NT‐proBNP N‐terminal pro‐B‐type natriuretic peptide) remain the gold standard against which other biomarkers are compared in HF, novel biomarkers associated with HF and HFpEF have emerged.^4^, ^5^ Many previous biomarker studies in HF have used single‐ or oligo‐marker approaches with varying methodology and statistic robustness, typically including only a minority of patients with AF.^6^, ^7^, ^8^, ^9^, ^10^, ^11^, ^12^, ^13^ The primary outcome of these trials has predominantly been incident HF rather than hospitalization for HF (HHF), thus resulting in a paucity of biomarker data about HHF in the AF population.^7^, ^11^, ^12^, ^13^, ^14^ Although many of the mechanisms, and therefore biomarkers, in the 2 populations likely are similar, some differences are expected. However, prior studies have not comprehensively characterized circulating biomarkers associated with HHF in patients with AF or examined whether biomarker profiles differ between patients with AF with HFpEF and HFrEF. The aim of this study was therefore to screen for plasma biomarkers associated with HHF in patients with AF and to compare biomarker profiles of AF in patients with HFpEF and HFrEF to better understand the risk for HHF and the underlying mechanisms and potential differences between the 2 subtypes of HF.

## Methods

The data that support the findings of this study are available from the corresponding author upon reasonable request.

### Patient Population and Study Design

**Figure 1:**
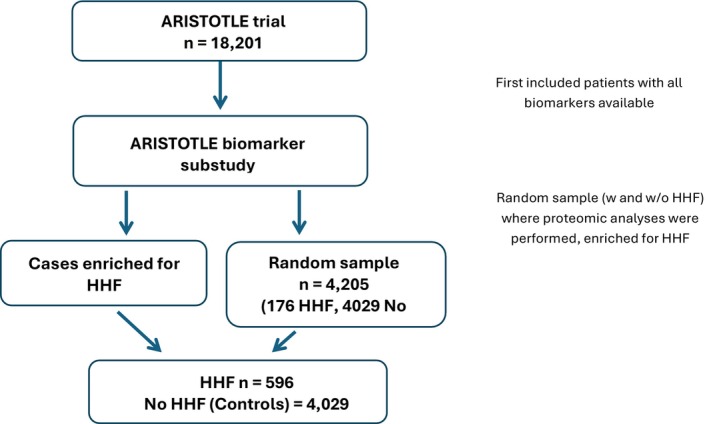
Study population selection for analyses of biomarkers associated with HHF in AF. AF indicates atrial fibrillation; and HHF, hospitalization for heart failure.

The ARISTOTLE (Apixaban for Reduction in Stroke and Other Thromboembolic Events in Atrial Fibrillation) trial enrolled 18 201 patients with AF and at least 2 CHADS_2_ risk factor for stroke or systemic embolism.^15^ The patients were randomized to receive apixaban or warfarin for prevention of stroke or systemic embolism. The median follow‐up time was 1.9 years. The present study population was derived from the ARISTOTLE trial biomarker substudy (N=14 757) from which a random sample representative of the trial population was selected (n=4205) that was enriched for cases not selected in the random sample (n=420) (Figure 1). Thus, using this case–cohort design, the present study cohort consisted of 596 cases with hospitalizations for HF during follow‐up and 4029 controls without hospitalization for HF during follow‐up. In accordance with the case–cohort design, patients with other events could be included in the random sample of controls.

Of the random sample of 4205, a total of 1211 patients had known HF and left ventricular systolic function at baseline, measured by echocardiography, contrast or radionuclide ventriculography, or magnetic resonance imaging. These patients formed the subset used for comparative analysis of biomarker profiles between HFpEF and HFrEF. All patients with prevalent HF at baseline were included, irrespective of whether they later experienced HHF during follow‐up; thus, these analyses reflect cross‐sectional differences in biomarker profiles according to prevalent HF at baseline and are distinct from the analyses of incident HHF. Patients were categorized into 3 groups: HFpEF (n=649), defined as symptomatic HF and an LVEF >40% (or if a numerical LVEF value was not available, a report of normal or only mildly reduced left ventricular systolic function); HFrEF (n=562), defined as an LVEF ≤40% (or if a numerical LVEF value was not available, a report of moderately or severely reduced left ventricular systolic dysfunction) regardless of symptoms of HF; and no HF (n=1917), defined by no symptoms of HF and a LVEF >40% (or if a numerical LVEF value was not available, a report of normal or only mildly reduced left ventricular systolic function).

Approval by the appropriate ethics committees was obtained at all sites and countries, and all patients provided written informed consent.

### Outcome Definition

The outcome for this multimarker study was HHF. Information on HHF during follow‐up was collected from the trial case report forms according to the primary reason for admission, as determined by trial investigators. Information on HF status at baseline was collected from the trial case report form at randomization.

### Biomarker Analyses

Analyses were performed using a high‐throughput technique using the OLINK Proteomics Multiplex CVD II, CVD III, and Inflammation panels (Thermo Fisher Scientific, Waltham, MA). Each panel allowed 92 biomarkers to be measured simultaneously by the binding of antibody‐paired DNA sequences to the target protein, resulting in DNA reporter sequences that were amplified by real‐time polymerase chain reaction.^16^ The proximity extension assays (PEAs) have shown high reproducibility and repeatability, with mean intra‐ and interassay coefficients of variation of ≈8% and 12%, respectively, and an average intersite variation of around 15% and have been shown to have an adequate concordance with conventional immunoassays.^16^, ^17^ The resulting, arbitrary, normalized protein expression levels, were log_2_‐transformed. The proteomic analyses were performed at the Clinical Biomarkers Facility (today called Affinity Proteomics), Science for Life Laboratory, Uppsala University (Uppsala, Sweden). In total, using the proteomics panels, the measurement of 276 preselected proteins associated with cardiovascular disease and inflammation was possible. Of these analyzed biomarkers, 10 were available on >1 panel, resulting in 266 unique biomarkers with the PEA analyses.

Additionally, plasma concentrations of hs‐cTnT (high‐sensitivity cardiac troponin T), NT‐proBNP, and GDF‐15 (growth differentiation factor 15) (precommercial assay) were determined by Roche immunoassays using a Cobas Analytics e601 (Roche Diagnostics, Penzberg, Germany) and interleukin‐6 high‐sensitivity sandwich ELISA immunoassays (R&D Systems Inc, Minneapolis, MN). Cystatin C was analyzed with the ARCHITECT system ci8200 (Abbott Laboratories, Abbott Park, IL) using the particle‐enhanced turbidimetric immunoassay from Gentian (Moss, Norway). All conventional analyses were performed at the Uppsala Clinical Research Center laboratory at Uppsala University and have been detailed previously.^18^, ^19^, ^20^, ^21^ Plasma creatinine was measured centrally, and estimated glomerular filtration rate was calculated using the Chronic Kidney Disease Epidemiology Collaboration equation.^22^

### Statistical Analysis

Baseline characteristics are summarized, within subgroups, by the sample median and interquartile range for continuous variables and proportions and counts for categorical variables.

The association between clinical variables (randomized treatment, age, sex, body mass index, smoking, hypertension, diabetes, hemoglobin, and previous myocardial infarction, stroke/transient ischemic attack, peripheral artery disease, HF, and bleeding) and biomarkers and the hazard of HHF was simultaneously evaluated by fitting a random survival forest. A permutation variable importance measure was used to rank the variables’ importance for predicting HHF. The random survival forest^23^ and Boruta algorithm^24^ approach was selected as a robust, nonparametric framework capable of handling nonlinear interactions and simultaneously assessing all biomarkers and clinical variables. A total of 268 biomarkers were analyzed with the random survival forest algorithm, 5 analyzed by conventional immunoassays (NT‐proBNP, hs‐cTnT, GDF‐15, interleukin‐6, and renal function) together with 266 biomarkers analyzed with PEA, excluding 3 (PEA biomarker duplicates that were analyzed by conventional analyses). The top 50 variables according to the variable importance are presented graphically.

Feature selection was performed using the Boruta algorithm. In combination with the random survival forest, Boruta simultaneously considered all candidate biomarkers (n=268) together with clinical variables. Briefly, random survival forests were repeatedly run with permuted copies of each variable added as noise. Variables were either rejected if no better than their permuted versions or confirmed as important. The process continued until all variables were decided or a maximum of 100 runs was reached. Remaining undecided variables were labeled tentative.

Furthermore, to evaluate each biomarker in isolation, Cox regression analyses, including each biomarker as a continuous variable, were performed adjusting for clinical variables (HF, age, sex, body mass index, smoking, hypertension, diabetes, prior myocardial infarction, prior stroke/transient ischemic attack, peripheral artery disease, and randomized treatment) and renal function (cystatin C) in Cox model 1. Cox model 2 was further adjusted for the cardiac biomarkers NT‐proBNP and hs‐cTnT. The results are presented as hazard ratios comparing the third and first sample quartile of the respective biomarker to put them on a somewhat equal scale. On the inflammation PEA panel, 16 of the proteins had >80% of the measurements below the limit of detection and were therefore not included in the Cox regression models. Therefore, the total number of biomarkers analyzed using Cox analyses was 255. The top 50 biomarkers are presented according to the *P* value for the respective biomarker.

In patients with HF at study entry, the association of the PEA markers and type of HF at baseline (ie, HFrEF or HFpEF) was assessed with a Wilcoxon–Mann–Whitney test for each marker. A Bonferroni–Holm procedure was used for multiplicity adjustment of the *P* values. For the markers passing the multiplicity adjustment (*P*<0.05 after adjustment), the association was denoted significant, and the distribution was shown for the groups using empirical cumulative distribution function plots.

## Results

Baseline characteristics of the 596 patients with HHF during follow‐up and the 4029 controls are detailed in Table 1 and for subgroups defined by baseline HFpEF, HFrEF, and no HF status are shown in Table 2. The age distribution was similar between the group with HHF and the controls. However, the HHF group exhibited a higher proportion of a history of HF, diabetes, atherosclerotic disease, and treatment with angiotensin‐converting enzyme (ACE) inhibitors (Table 1). Additionally, these patients had higher baseline levels of cardiac markers (hs‐cTnT and NT‐proBNP), markers of inflammation (C‐reactive protein and interleukin‐6), and GDF‐15.

**Table 1:** Baseline Characteristics and Concentration of Established Biomarkers

Baseline characteristics	Random sample N=4029	HHF N=596
Age, y	70.0 (63–76)	71.0 (64–77)
Women	1477 (36.7)	218 (36.6)
Body mass index	28.6 (25.3–32.7)	28.5 (25.0–33.4)
Current smoker	352 (8.7)	58 (9.7)
Hypertension	3526 (87.5)	521 (87.4)
Diabetes	1003 (24.9)	197 (33.1)
Prior myocardial infarction	489 (12.1)	153 (25.7)
Prior stroke/transient ischemic attack	737 (18.3)	118 (19.8)
Peripheral artery disease	190 (4.7)	51 (8.6)
HF	1197 (29.7)	346 (58.1)
β blockers	2586 (64.2)	422 (70.8)
Angiotensin‐converting enzyme inhibitors	1965 (48.8)	375 (62.9)
NT‐proBNP, ng/L	681.0 (358.0–1206.0)	1373.5 (811.8–2301.2)
hs‐cTnT, ng/L	10.6 (7.4–16.2)	16.6 (11.0–26.1)
GDF‐15, ng/L	1352.0 (962.0–2037.0)	1905.0 (1321.5–3025.8)
Cystatin C, mg/L	1.0 (0.8–1.2)	1.1 (0.9–1.4)
Interleukin‐6, ng/L	2.3 (1.5–3.9)	3.6 (2.1–5.8)
C‐reactive protein, mg/L	2.2 (1.0–4.6)	3.3 (1.5–7.3)
CKD‐EPI (Cystatin C), mL/min	75.5 (57.6–97.1)	62.1 (45.2–80.6)

Continuous variables are presented as median (interquartile range), and categorical variables are presented as number (percentage). CKD‐EPI indicates Chronic Kidney Disease Epidemiology Collaboration; GDF‐15, growth differentiation factor 15; HF, heart failure; HHF, hospitalization for heart failure; hs‐cTnT, high‐sensitivity cardiac troponin T; and NT‐proBNP, N‐terminal pro‐B‐type natriuretic peptide.

**Table 2:** Baseline Characteristics of Participants With and Without Prevalent HF (Either HFpEF or HFrEF) at Randomization

Variable	No HF (N=1917)	HFpEF (N=649)	HFrEF (N=562)
Age, y	70.0 (63.0–76.0)	69.0 (62.0–74.0)	67.0 (59.0–73.0)
Women	705 (36.8)	291 (44.8)	116 (20.6)
Body mass index	28.7 (25.5–32.7)	29.4 (25.8–33.6)	28.0 (24.9–32.6)
Current smoker	158 (8.2)	89 (9.1)	71 (12.6)
Hypertension	1732 (90.3)	566 (87.2)	438 (77.9)
Diabetes	504 (26.3)	157 (24.2)	142 (25.3)
Prior myocardial infarction	203 (10.6)	104 (16.0)	135 (24.0)
Prior stroke/transient ischemic attack	368 (19.2)	108 (16.6)	80 (14.2)
Peripheral artery disease	90 (4.7)	40 (6.2)	34 (6.0)
Symptomatic HF within 3 mo	0 (0.0)	649 (100.0)	374 (66.5)
β blockers	1241 (64.7)	450 (69.3)	432 (76.9)
Angiotensin‐converting enzyme inhibitors	829 (43.2)	376 (57.9)	378 (67.3)
NT‐proBNP, ng/L	618.0 (323.5–1057.0)	746.0 (399.5–1340.5)	1060.5 (571.8–2052.2)
hs‐cTnT, ng/L	9.9 (7.1–14.7)	11.1 (7.7–17.3)	14.1 (9.5–22.1)
GDF‐15, ng/L	1315.0 (946.0–1939.0)	1370.0 (951.0–2052.0)	1579.0 (1080.2–2429.8)
Cystatin C, mg/L	0.9 (0.8–1.1)	1.0 (0.8–1.2)	1.1 (0.9–1.3)
Interleukin‐6, ng/L	2.1 (1.4–3.5)	2.4 (1.5–4.2)	2.9 (1.8–5.0)
C‐reactive protein, mg/L	2.0 (0.9–4.2)	2.6 (1.1–6.0)	2.4 (1.1–5.1)
CKD‐EPI (Cystatin C), mL/min	77.9 (60.4–98.8)	74.5 (54.9–93.5)	67.2 (50.8–88.9)

Continuous variables are presented as median (interquartile range), and categorical variables are presented as number (percentage). CKD‐EPI indicates Chronic Kidney Disease Epidemiology Collaboration; GDF‐15, growth differentiation factor 15; HF, heart failure; HFpEF, heart failure with preserved ejection fraction; HFrEF, heart failure with reduced ejection fraction; hs‐cTnT, high‐sensitivity cardiac troponin T; and NT‐proBNP, N‐terminal pro‐B‐type natriuretic peptide.

### Biomarkers Associated With HHF

#### Random Survival Forest Analyses

**Figure 2:**
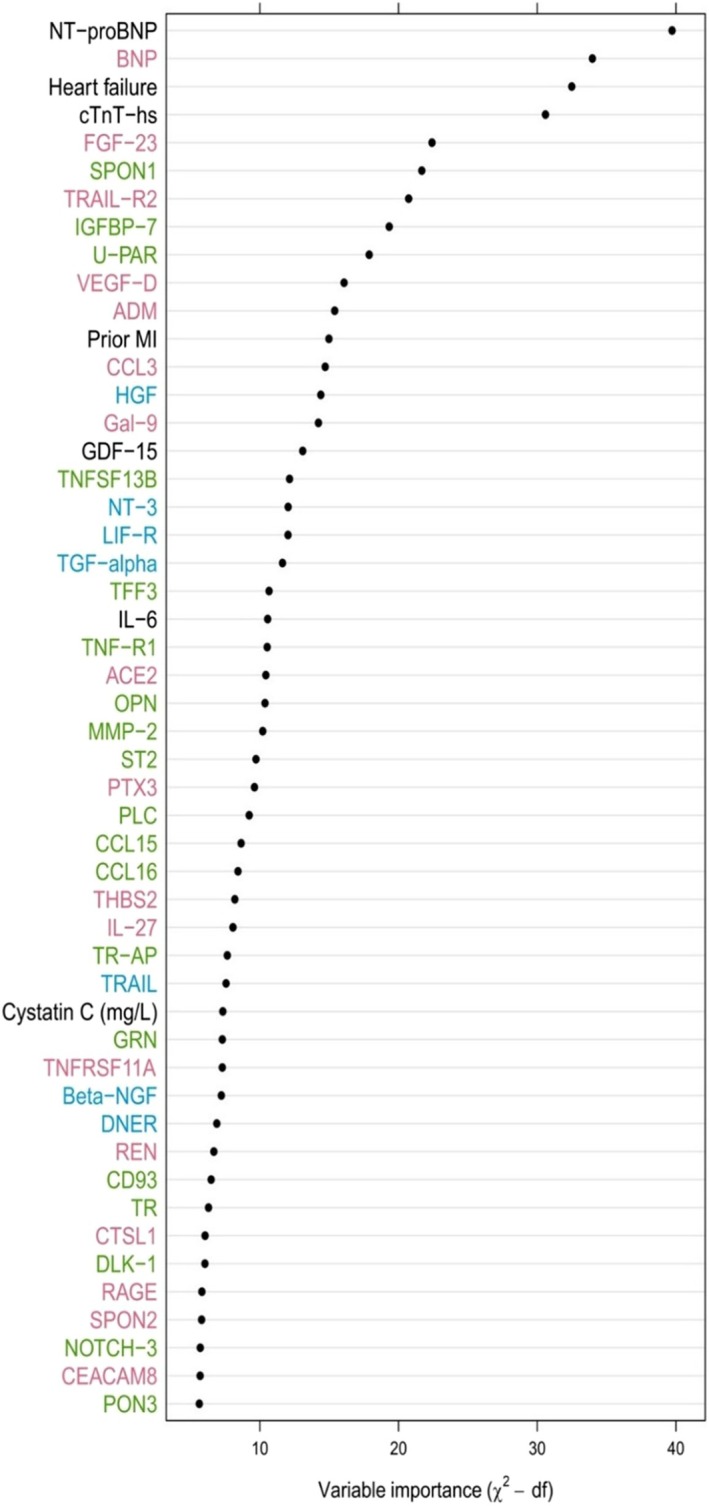
Variable importance of the top 50 biomarkers most strongly associated with HHF according to the random survival forest analysis. Red color indicates biomarkers analyzed on CVD II panel; green, biomarkers analyzed on CVD III panel; and blue, biomarkers analyzed on Inflammation panel. Biomarkers listed in black were analyzed with conventional immunoassays. ACE2 indicates angiotensin‐converting enzyme 2; ADM, pro‐adrenomedullin; Beta‐NGF, beta nerve‐growth factor; BNP, B‐type natriuretic peptide; CCL15, chemokine ligand 15; CCL16, chemokine ligand 16; CCL3, chemokine ligand 3; CD93, complement component C1q receptor; CEACAM8, carcinoembryonic antigenrealted cell adhesion molecule 8; CTSL1, cathepsin L1; CVD, cardiovascular disease; DLK‐1, protein delta homolog 1; DNER, delta and notch‐like epidermal growth factor‐related receptor; FGF‐23, fibroblast growth factor 23; Gal‐9, galectin‐9; GDF‐15, growth differentiation factor 15; GRN, granulin precursor; HGF, hepatocyte growth factor; HHF, hospitalization for heart failure; hs‐cTnT, high‐sensitivity cardiac troponin T; IGFBP‐7, insulin‐like growth factor binding protein 7; IL‐6, interleukin‐6; IL‐27, interleukin‐27; LIF‐R, leukemia inhibitory factor receptor; MI, myocardial infarction; MMP2, matrix metalloproteinase‐2; NOTCH‐3, neurogenic locus notch homolog protein 3; NT‐3, neurotrophin‐3; NT‐proBNP, N‐terminal pro‐B‐type natriuretic peptide; OPN, osteopontin; PLC, perlecan; PON3, paraoxonase/lactonase 3; PTX3, pentraxin‐related protein; RAGE, receptor for advanced glycosylation end products; REN, renin; SPON1, spondin 1; SPON2, spondin 2; ST2, soluble interleukin 1 receptor‐like 1; TFF3, trefoil factor 3; TGF‐alpha, transforming growth factor‐α; THBS2, thrombospondin‐2; TNF‐R1, tumor necrosis factor receptor 1; TNFRSF11A; TNFRSF13B, tumor necrosis factor receptor superfamily member 13B; TR, transferrin receptor protein 1; TRA‐Ap, tarttrate‐resistant acid phosphatase type 5; TRAIL, tumor necrosis factor–related apoptosis‐inducing ligand; TRAIL‐R2, tumor necrosis factor–related apoptosis‐inducing ligand receptor 2; tumor necrosis factor superfamily member 11A; U‐PAR, urokinase‐ type plasminogen activator receptor; and VEGF‐D, vascular endothelial growth factor D.

Of a total of 268 biomarkers and 13 clinical variables, the 50 variables with strongest associations with HHF according to the random survival forest analysis are shown in Figure 2. NT‐proBNP had the strongest positive association with HHF, followed by BNP, history of HF, hs‐cTnT, FGF‐23 (fibroblast growth factor 23), spondin 1 and tumor necrosis factor–related apoptosis‐inducing ligand receptor 2. The importance of these biomarkers was also confirmed in the corresponding Boruta analysis. In total, the Boruta analysis confirmed the importance of 42 biomarkers (Table 3).

**Table 3:** Random Survival Forest Ranking and Cox Regression Multivariate Associations of Biomarkers With the Highest Association With HHF

Biomarker	Random forest ranking	Model 1		Model 2		Boruta
		Hazard ratio (95% CI)	*P* value	Hazard ratio (95% CI)	*P* value	
NT‐proBNP*	1	2.325 (1.977–2.735)	<1.0e‐16	2.068 (1.759–2.432)	<1.0e‐16	Confirmed
BNP*	2	2.256 (1.948–2.613)	<1.0e‐16	1.572 (1.246–1.982)	1.3e−04	Confirmed
hs‐cTnT*	3	1.644 (1.418–1.905)	4.5e−11	1.346 (1.148–1.579)	2.5e−04	Confirmed
FGF‐23*	4	1.434 (1.319–1.558)	<1.0e‐16	1.264 (1.153–1.385)	5.5e−07	Confirmed
Spondin 1*	5	1.385 (1.273–1.507)	3.6e−14	1.211 (1.099–1.334)	1.0e−04	Confirmed
TRAIL‐R2	6	1.292 (1.175–1.421)	1.1e−07	1.190 (1.070–1.323)	1.3e−03	Confirmed
IGFBP‐7*	7	1.443 (1.307–1.594)	4.6e−13	1.378 (1.127–1.393)	2.9e−05	Confirmed
U‐PAR*	8	1.716 (1.465–2.011)	2.4e−11	1.406 (1.194–1.655)	4.2e−05	Confirmed
VEGF‐D	9	1.707 (1.450–2.008)	1.2e−10	1.284 (1.080–1.526)	4.6e−03	Confirmed
Adrenomedullin	10	1.524 (1.241–1.873)	5.9e−05	1.202 (1.032–1.399)	1.8e−02	Confirmed
CCL3	11	1.194 (1.115–1.278)	4.0e−07	1.147 (1.062–1.238)	4.6e−04	Confirmed
HGF	12	1.265 (1.174–1.363)	7.2e−10	1.152 (1.056–1.257)	1.4e−03	Confirmed
Galectin‐9	13	1.407 (1.175–1.686)	2.1e−04	1.295 (1.084–1.547)	4.4e−03	Confirmed
GDF‐15	14	1.624 (1.399–1.885)	1.9e−10	1.289 (1.103–1.398)	1.7e−03	Confirmed
TNFRSF13B	15	1.096 (0.960–1.251)	1.8e−01	1.242 (0.993–1.225)	3.4e−04	Confirmed
Neurotrophin 3	16	1.232 (1.104–1.375)	1.9e−04	1.122 (1.022–1.233)	1.6e−02	Confirmed
LIF‐R	17	1.583 (1.391–1.802)	3.4e−12	1.260 (1.093–1.451)	1.4e−03	Confirmed
TGFA	18	1.298 (1.155–1.459)	1.3e−05	1.163 (1.024–1.322)	2.0e−02	Confirmed
TFF3	19	1.299 (1.151–1.467)	2.3e−05	1.188 (1.041–1.355)	1.0e−02	Confirmed
Interleukin‐6	20	1.419 (1.275–1.580)	1.7e−10	1.188 (1.056–1.337)	4.1e−03	Confirmed
TNF‐R1	21	1.325 (1.113–1.577)	1.6e−03	1.132 (0.958–1.338)	1.5e−01	Confirmed
ACE2	22	1.420 (1.271–1.586)	4.9e−10	1.218 (1.077–1.378)	1.7e−03	Confirmed
Osteopontin *	23	1.496 (1.302–1.718)	1.2e−08	1.313 (1.139–1.514)	1.8e−04	Confirmed
MMP2	24	1.644 (1.418–1.905)	4.5e−11	1.286 (1.116–1.482)	5.2e−04	Confirmed
ST2	25	1.482 (1.319–1.665)	3.9e−11	1.215 (1.083–1.364)	8.8e−04	Confirmed
PTX3*	26	1.502 (1.333–1.693)	2.7e−11	1.298 (1.146–1.469)	4.0e−05	Confirmed
Phospholipase C	27	1.370 (1.145–1.639)	5.7e−04	1.127 (0.952–1.334)	1.7e−01	Confirmed
CCL15	28	1.239 (1.095–1.402)	6.8e−04	1.164 (1.021–1.327)	2.3e−02	Confirmed
CCL16	29	1.118 (0.999–1.252)	5.3e−02	1.096 (0.977–1.229)	1.2e−01	Confirmed
Thrombospondin 2	30	1.498 (1.273–1.763)	1.2e−06	1.175 (0.979–1.411)	8.4e−02	Confirmed
Interleukin‐27	31	1.383 (1.221–1.567)	3.5e−07	1.213 (1.058–1.391)	5.6e−03	Confirmed
TRAP	32	0.772 (0.693–0.860)	2.6e−06	0.865 (0.766–0.977)	2.0e−02	Confirmed
TRAIL	33	0.736 (0.652–0.831)	7.6e−07	0.820 (0.721–0.932)	2.4e−03	Confirmed
Cystatin C	34	1.663 (1.468–1.883)	1.2e−15	1.130 (0.980–1.302)	9.3e−02	Confirmed
GRN	35	1.326 (1.166–1.508)	1.7e−05	1.272 (1.113–1.453)	4.1e−04	Confirmed
DNER	38	0.804 (0.720–0.898)	1.0e−04	0.861 (0.762–0.974)	1.7e−02	Confirmed
Renin	39	1.108 (0.963–1.274)	1.5e−01	1.228 (1.062–1.419)	5.6e−03	Confirmed
TR*	41	1.376 (1.231–1.539)	2.2e−08	1.262 (1.120–1.421)	1.3e−04	Confirmed
Cathepsin L1	42	1.302 (1.150–1.474)	3.0e−05	1.184 (1.034–1.354)	1.4e−02	Confirmed
PON3	48	0.838 (0.757–0.928)	6.7e−04	0.860 (0.764–0.968)	1.3e−02	Confirmed
GIF	49	0.809 (0.727–0.900)	1.0e−04	0.845 (0.754–0.948)	3.9e−03	Confirmed
TWEAK	51	0.882 (0.801–0.971)	1.0e−02	0.899 (0.813–0.995)	3.9e−02	Confirmed

Biomarkers confirmed by Boruta analysis included, ranked according to random survival forest variable importance. Biomarkers marked with Hazard ratios per interquartile range. Biomarkers in Model 1: Cox regression analysis model adjusted for clinical characteristics: age, sex, body mass index, smoking, hypertension, diabetes, prior myocardial infarction, prior stroke/transient ischemic attack, prior peripheral artery disease, prior HF, randomized treatment. Model 2: model 1+adjustment for renal function and NT‐proBNP + hs‐cTnT. ACE2 indicates angiotensin‐converting enzyme 2; BNP, B‐type natriuretic peptide; CCL3, chemokine ligand 3; CCL15, chemokine ligand 15; CCL16, chemokine ligand 16; DNER, delta and notch‐like epidermal growth factor‐related receptor; FGF‐23, fibroblast growth factor 23; GFD‐15, growth differentiation factor 15; GIF, cobalamin‐binding intrinsic factor; GRN, granulin precursor; HF, heart failure; HGF, hepatocyte growth factor; HHF, hospitalization for heart failure; hs‐cTnT, high‐sensitivity cardiac troponin T; IGFBP‐7, insulin‐like growth factor binding protein 7; LIF‐R, leukemia inhibitory factor receptor; MMP2, matrix metalloproteinase‐2; NT‐proBNP, N‐terminal pro‐B‐type natriuretic peptide; PON3, paraoxonase/lactonase 3; PTX3, pentraxin‐related protein; TFF3, trefoil factor 3; TGFA, transforming growth factor‐α; TNF‐R1, tumor necrosis factor receptor 1; TNFRSF13B, tumor necrosis factor receptor superfamily member 13B; TR, transferrin receptor protein 1; TRAIL, tumor necrosis factor–related apoptosis‐inducing ligand; TRAIL‐R2, tumor necrosis factor–related apoptosis‐inducing ligand receptor 2; TRAP, CD40 ligand; TWEAK, tumor necrosis factor ligand superfamily member 12; U‐PAR, urokinase‐ type plasminogen activator receptor; and VEGF‐D, vascular endothelial growth factor D.

* Biomarkers *P*≤0.00027 (multiplicity adjusted) in adjusted Cox regression model 2 and confirmed in Boruta analyses.

#### Cox Regression Analyses

**Figure 3:**
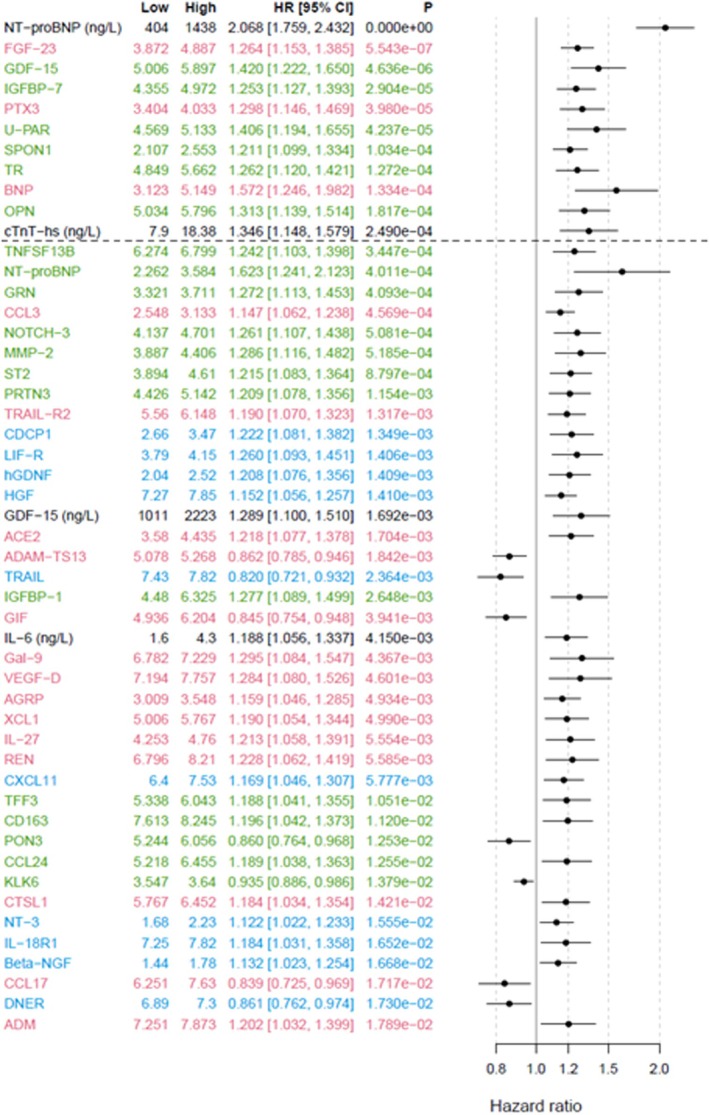
Forest plot of the top 50 biomarkers associated with HHF according to adjusted Cox regression analysis (by *P* value). The dashed line indicates the multiplicity‐adjusted significance threshold (*P*≤0.000266) in the Cox model; all biomarkers positioned above this line remained statistically significant after adjustment. Red color indicates biomarkers analyzed on CVD II panel; green, biomarkers analyzed on CVD III panel; and blue, biomarkers analyzed on Inflammation panel. Biomarkers listed in black were analyzed with conventional immunoassays. HHF indicates hospitalization for heart failure.

According to the Cox regression analyses adjusted for clinical characteristics and renal function (Cox model 1), of 255 biomarkers, 46% (n=116) were associated with HHF. When further adjusting for the 2 cardiac biomarkers (NT‐proBNP and hs‐cTnT) (Cox model 2), 27% (n=68) of the biomarkers remained associated (*P*<0.05) with HHF (top 50 shown in Figure 3). Among the 42 biomarkers confirmed in the Boruta analysis, 37 biomarkers were associated (*P*<0.05) with the outcome (Table 3). When adjusting for multiplicity, 10 biomarkers remained significant (*P*≤0.0002665); NT‐proBNP, BNP, hs‐cTnT, FGF‐23, spondin 1, IGFBP‐7 (insulin‐like growth factor binding protein 7), soluble urokinase‐ type plasminogen activator receptor, osteopontin, PTX3 (pentraxin‐related protein), and TR (transferrin receptor protein 1).

### Biomarker Profiles in HFpEF Versus HFrEF in Patients With Prevalent HF

**Figure 4:**
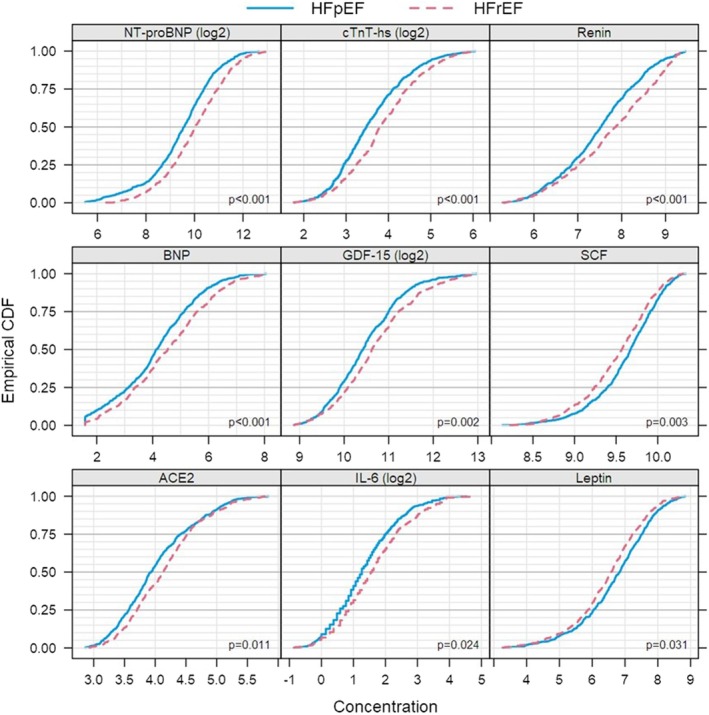
Empirical cumulative distribution function of biomarkers according to HF subtype. HF indicates heart failure.

In a distinct analysis comparing HFpEF with HFrEF profiles in patients with prevalent HF at baseline, of 268 biomarkers, 53 biomarkers differed between HFpEF and HFrEF in patients with prevalent HF (Table S1). After adjustment for multiplicity, 9 biomarkers were significantly different (*P*<0.05) between HFpEF and HFrEF (Table 4). In HFpEF, the levels of SCF (stem cell factor) and leptin were higher than in HFrEF, whereas levels of NT‐proBNP, BNP, hs‐cTnT, renin, GDF‐15, ACE‐2, and interleukin‐6 were higher in HFrEF (Figure 4). In sensitivity analyses, the results for renin and ACE2 were materially unaltered when adjusted for treatment with ACE inhibitors/angiotensin receptor blockers and diuretics within 30 days before randomization. A summary of these biomarkers is shown in Table 4.

**Table 4:** Summary of Biomarkers With the Strongest Difference Between HFpEF and HFrEF

Protein	Median HFpEF	Median HFrEF	Relative median HFpEF	Relative difference of median HFrEF vs HFpEF	*P* value	*P* value adjusted
NT‐proBNP (log_2_)*	953 527 538	100 505 249	1	142 924 131	2.5E‐12	6.8E‐10
Renin*	751 158 293	787 313 518	1	128 480 752	1.6E‐07	4.2E‐05
hs‐cTnT (log_2_)*	347 248 777	382 272 114	1	127 476 682	1.0E‐09	2.7E‐07
BNP*	410 283 075	456 128 965	1	137 407 323	2.4E‐07	6.5E‐05
SCF†	967 360 325	956 611 021	1	0.92 819 958	1.2E‐05	0.0032
GDF‐15 (log_2_)*	104 199 602	106 247 943	1	115 255 382	5.7E‐06	0.0015
ACE2*	392 957 549	412 990 073	1	114 895 734	4.1E‐05	0.011
Leptin†	684 624 058	657 601 232	1	0.82 918 835	0.00012	0.031
Interleukin‐6 (log_2_)*	126 303 441	15 360 529	1	120 833 333	9.0E‐05	0.0236

ACE2 indicates angiotensin‐converting enzyme 2; BNP, B‐type natriuretic peptide; GDF‐15, growth differentiation factor 15; HFpEF, heart failure with preserved ejection fraction; HFrEF, heart failure with reduced ejection fraction; SCF, stem cell factor; hs‐cTnT, high‐sensitivity cardiac troponin T; and NT‐proBNP, N‐terminal pro‐B‐type natriuretic peptide.

* Biomarker with higher concentrations in HFrEF.

^†^ Biomarkers with higher concentrations in HFpEF. Biomarker levels are reported as normalized protein expression units and are relative measures of protein expression and do not represent absolute concentrations. The relative difference between the medians is calculated as 2^(difference of medians on log2 scale)^.

## Discussion

In the present study, we conducted a comprehensive multiplex screening of 268 plasma proteins to identify biomarkers and related pathways associated with subsequent HHF in patients with AF, with and without HF at entry. We identified 10 biomarkers being significantly associated with HHF after multiplicity adjustment: NT‐proBNP, BNP, hs‐cTnT, FGF‐23, spondin1, IGFBP‐7, urokinase receptor, osteopontin, PTX3, and TR.

In a subset of patients with known HF at baseline, we also examined baseline differences in biomarker levels between those fulfilling the criteria for HFrEF and HFpEF. Our findings revealed significant differences in biomarker levels between the 2 HF subtypes. Specifically, we observed higher levels of SCF and leptin in patients with HFpEF, while NT‐proBNP, BNP, hs‐cTnT, renin, GDF‐15, ACE‐2, and interleukin‐6 levels were higher in patients with HFrEF.

### Biomarkers Associated With HHF in AF

The natriuretic peptides (NT‐proBNP, BNP) alongside cardiac troponins (hs‐cTnT) have been the most extensively investigated and clinically used cardiac markers for assessing HF diagnosis, worsening, and prognosis.^2^ In the present study, both natriuretic peptides and cardiac troponins emerged as the biomarkers with the strongest association with the risk of HHF in AF. NT‐proBNP, BNP, and hs‐cTnT have previously demonstrated strong associations with other adverse cardiovascular outcomes in AF, indicating their relevance for disease prognosis.^25^, ^26^, ^27^ However, there is a need for better understanding of the processes involved in patients with AF and the risk for HHF beyond the information provided by natriuretic peptides and cardiac troponins. Thus, the evaluation of other identified biomarkers and their related pathways are of high importance to elucidate.

The 7 other biomarkers, FGF‐23, spondin 1, IGFBP‐7, urokinase‐type plasminogen activator receptor, osteopontin, PTX3, and TR, which displayed the strongest association with the risk of HHF, have consistently demonstrated similar strong associations in several other studies examining incident and worsening HF mainly in populations with a low degree of AF, as well as in patients with high AF prevalence.^11^, ^14^, ^28^, ^29^ Furthermore, there are data from Mendelian randomization studies suggesting that the inflammatory markers spondin 1, IGFBP‐7, and the peptide hormone FGF‐23, which regulates phosphate and calcium balance, have a causal relationship with HF. Specifically, spondin 1 and IGFBP‐7 have a potential causal association with left ventricular size and function, while FGF‐23 possibly exerts protective effects instead.^29^, ^30^ Although 6 biomarkers were associated with reduced risk of HHF (CD40 ligand, tumor necrosis factor ligand superfamily member 10, delta and notch‐like epidermal growth factor‐related receptor, serum paraoxonase/lactonase 3, cobalamin‐binding intrinsic factor, and tumor necrosis factor ligand superfamily member 12), they did not reach statistical significance after multiplicity adjustment (Table 3).

Our results from this multimarker study expand the understanding by extending previously described findings also to an AF population. Considered together, our findings strengthen the evidence for the involvement of these biomarkers and their corresponding processes in HF.

### Biomarker Profiles in HFpEF Versus HFrEF

Although natriuretic peptides can be used as a diagnostic tool in both the HF types HFpEF and HFrEF, patients with HFpEF generally exhibit lower levels of natriuretic peptides compared with patients with HFrEF.^31^ This is believed to result from a lesser degree of myocardial wall stress in HFpEF.^32^ Similarly, higher levels of cardiac troponins have been seen in patients with HFrEF, signifying myocardial damage and loss of cardiomyocytes.^3^, ^33^ The results of the present study confirm these findings, with NT‐proBNP, BNP, and hs‐cTnT levels being significantly higher in patients with HFrEF.

GDF‐15 is expressed in states of inflammatory and oxidative stress and exerts anti‐inflammatory actions, and plasma levels do not seem to be influenced by AF.^34^, ^35^ Higher levels of GDF‐15 were associated with both incident HFpEF and HFrEF in a cohort were only a minority had AF.^36^ In our study, significantly higher levels of GDF‐15 were seen in HFrEF as compared with HFpEF. In contrast, prior data did not show any difference in protein levels between the 2 HF subtypes.^36^ The larger sample size of the present study and differences in patient population might account for some differences between the results of the 2 studies.

Another marker of inflammation is interleukin‐6, which in prior studies have been associated with both HFpEF and HFrEF.^37^, ^38^ Our study revealed higher levels of interleukin‐6 in HFrEF, consistent with previous findings of elevated interleukin‐6 concentrations in this HF subtype.^28^

Renin acts as an activator, and ACE‐2 as a counterregulator, of the renin–angiotensin–aldosterone system that regulates volume and electrolyte balance and systemic vascular resistance and is central in HF pathophysiology.^39^ While HF and some antihypertensive medications can upregulate renin via renal feedback mechanisms, they do not seem to affect ACE‐2 levels.^40^, ^41^ Renin levels were significantly higher in patients with HFrEF, even when adjusted for HF medication, and perhaps signify a stronger renin–angiotensin–aldosterone system activation in HFrEF. Similarly, higher ACE‐2 activation has previously been observed in HFrEF, and our study further demonstrates higher ACE‐2 levels in HFrEF compared with HFpEF.^42^

The 2 biomarkers that were significantly higher in HFpEF were SCF and leptin. SCF plays an important role in vasculogenesis and tissue repair, and elevated SCF concentrations have been associated with lower risk of cardiovascular disease, including lower risk for HF.^43^, ^44^ On the other hand, increased leptin levels are seen in obesity as it acts as a satiety hormone while also inducing proinflammatory molecules.^45^ In a prior study in which approximately half of the cohort had AF, leptin concentrations increased in both HFpEF and HFrEF, and after adjustment for background variables, there was no leptin concentration difference between the 2 HF groups.^46^ Our study had a comparatively larger population, thus providing more adequate power for detecting group differences. Other possible explanations for the disparities between the 2 studies could be related to type of study population, comorbidities, and severity of disease. Although leptin has been shown to induce prohypertrophic and neurohormonal effects, experimental data also suggest that leptin may also exert protective effects against left ventricular hypertrophy, particularly in the setting of leptin deficiency or resistance.^47^ Importantly, the observed elevation in leptin levels among patients with HFpEF in the present study may reflect the strong association between HFpEF and obesity, a phenotype characterized by metabolic inflammation and adipose tissue–derived signaling, including hyperleptinemia.^48^, ^49^ However, the roles of SCF and leptin in the context of AF and HF remain incompletely understood, with both biomarkers possibly reflecting overlapping mechanisms related to inflammation, remodeling, and metabolic dysfunction. To our knowledge, the present study is the first to identify elevated SCF and leptin levels in HFpEF compared with HFrEF, shedding new light on their importance in these conditions.

**Figure 5:**
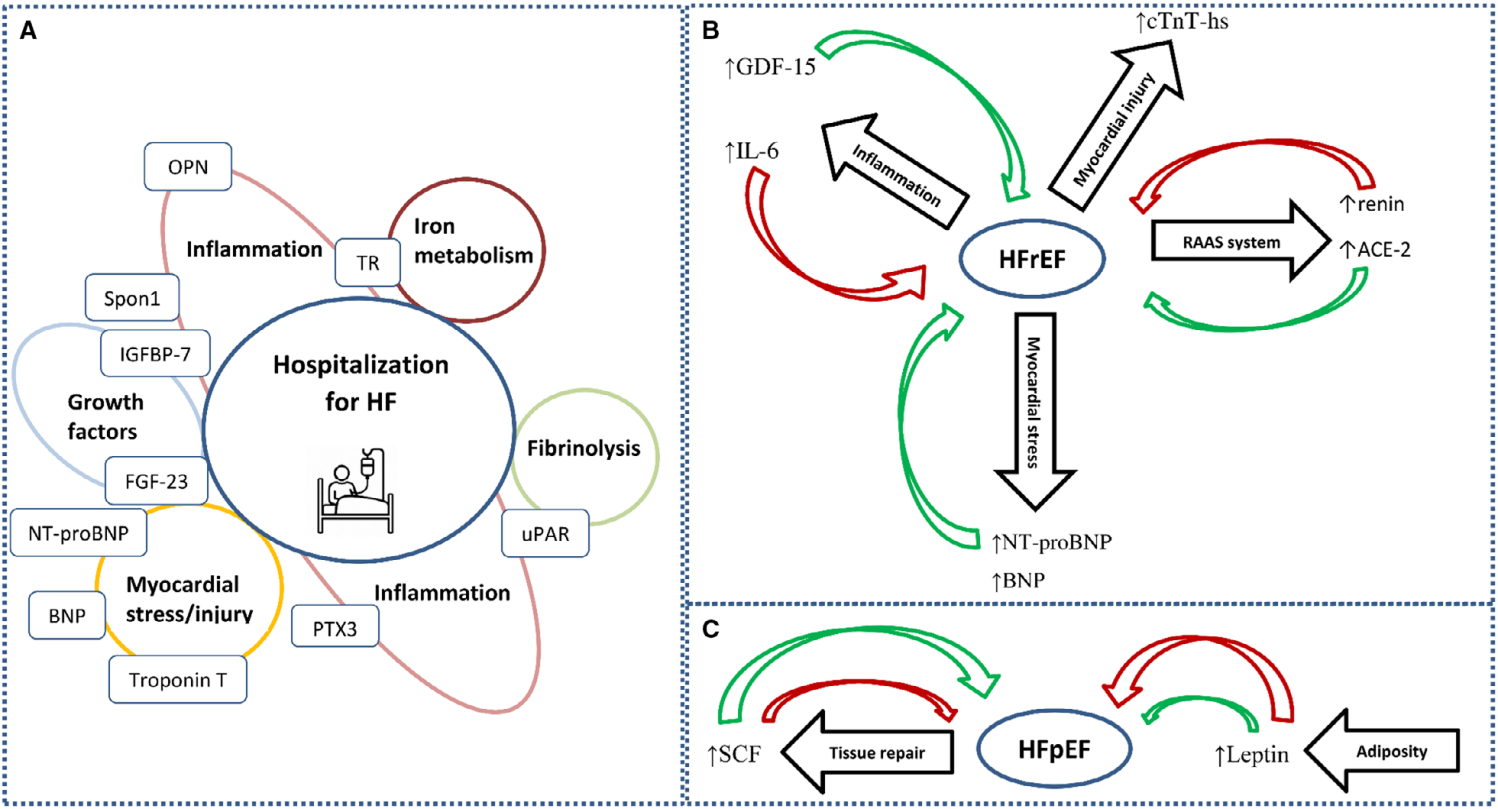
Conceptual summary of key findings. **A**, Biomarkers significantly associated with HHF in AF, grouped by predominant biological processes. **B**, Biomarkers higher in prevalent HFrEF with illustrated biological processes and their potential mechanistic links to disease. **C**, Biomarkers higher in prevalent HFpEF with illustrated biological processes and their potential mechanistic links to disease. Red arrows indicate predominantly harmful or maladaptive effects, while green arrows indicate predominantly protective or adaptive effects. AF indicates atrial fibrillation; HHF, hospitalization for heart failure; HFpEF, heart failure with preserved ejection fraction; and HFrEF, heart failure with reduced ejection fraction.

Taken together, our results provide valuable insights into the pathophysiological processes leading to HHF as well as the differing mechanisms underlying HFpEF and HFrEF (Figure 5). These findings might provide a better understanding of HF in the setting of patients with AF.

While studying a large number of studied proteins with robust statistical methods in a well‐controlled and large study population, there are several potential limitations to consider. The results reflect the study population; thus, variations in background characteristics, treatment, and HF type and severity might influence the findings. Moreover, the absence of adjudication for HHF could somewhat reduce outcome certainty. Even without adjusting the analyses for baseline heart rhythm, previously published data have shown that while individual biomarker levels may be influenced by rhythm status, the relationship between biomarkers and cardiovascular outcomes remains unaffected.^50^ It is also worth noting that AF can complicate the diagnosis of HHF, potentially affecting the reliability of our findings, as NT‐proBNP, a marker used to determine worsening of HF, is also elevated in patients with AF. Additionally, there was no standardization of LVEF measurement at baseline in the ARISTOTLE trial, raising the possibility of HFpEF/HFrEF misclassification. Due to the original trial protocol, HF with mildly reduced ejection fraction was included in the HFpEF category in this biomarker study. Although the definition of HFpEF has varied over the years and some mechanistic similarities exist between HFpEF and HF with mildly reduced ejection fraction, mechanistic differences are likely to be present and may impact the interpretation of our findings. Furthermore, our study focused solely on protein biomarkers, potentially overlooking contributions from other biomolecule classes such as lipids or nucleic acids, which could add to a more comprehensive understanding of the pathophysiology of HF. Thus, future studies incorporating multiomic approaches and larger patient cohorts are warranted to validate and expand upon our observations, ultimately paving the way for the development of more targeted and effective therapies for patients with HF.

## Conclusions

Of 268 evaluated biomarkers, this comprehensive screening study identified biomarkers representing cardiorenal dysfunction, growth factors, fibrinolysis, inflammation, and iron metabolism as being the most strongly associated with the risk of HHF in patients with AF with and without HF or a history of HF at baseline. In the subset of patients with HF at baseline, patients with HFrEF had higher levels of markers reflecting cardiorenal dysfunction, the renin–angiotensin–aldosterone system and oxidative stress/inflammation, whereas patients with HFpEF had higher levels of adipose tissue metabolism and vasculogenesis and tissue repair proteins. Our findings highlight important pathophysiological pathways involved in HHF and add valuable knowledge to further the understanding of the underlying biological mechanisms for development of HF in patients with AF, including the HF subtypes HFrEF and HFpEF.

## Sources of Funding

This work was supported by the Swedish Foundation for Strategic Research (Grant No. RB13‐0197); the Swedish Heart–Lung Foundation (Grant No. 20090183); and Science for Life Laboratory, Uppsala University (Uppsala, Sweden). The ARISTOTLE trial was funded by Bristol‐Myers Squibb (Princeton, NJ) and Pfizer Inc. (New York, NY), and coordinated by the Duke Clinical Research Institute (Durham, NC).

## Disclosures

J. Lindbäck reports institutional research grants from Boehringer Ingelheim, Bristol‐Myers Squibb/Pfizer. Dr Oldgren reports fees to his institution from AstraZeneca, Bayer HealthCare, Boehringer Ingelheim, Bristol‐Myers Squibb, Daiichi Sankyo, Pfizer, Portola, Roche Diagnostics, and Sanofi. Dr Alexander reports institutional research grants from Bayer, Boehringer Ingelheim, Bristol‐Myers Squibb, Cryolife, CSL Behring, Ferring, Glaxosmithkline, and XaTek; and consulting fees/honoraria from AbbVie, Bristol‐Myers Squibb, Cryolife, Glaxosmithkline, Pfizer, and Portola. Dr Siegbahn reports institutional research grants from AstraZeneca, Boehringer Ingelheim, Bristol‐Myers Squibb/Pfizer, GlaxoSmithKline, and Roche Diagostics; and consultancy fees from OLINK Proteomics. Dr Wallentin reports institutional research grants, consultancy fees, lecture fees, and travel support from Bristol‐Myers Squibb/Pfizer, AstraZeneca, GlaxoSmithKline, Boehringer Ingelheim; institutional research grants from Merck and Company and Roche; has received consultancy fees from Abbott; and holds 2 patents involving GDF‐15. Dr Hijazi reports employment by Thermo Fisher Scientific; lecture fees from Boehringer Ingelheim, Roche, Bristol‐Myers Squibb, and Pfizer; consulting fees from Merck Sharp and Dohme, Roche, Bristol‐Myers Squibb, and Pfizer; and research grants from the Swedish Heart–Lung Foundation. Dr Pol has no disclosures to report.

## Supporting information

Table S1
