# Supplementary material for: Plasma Biomarkers Associated With Heart Failure Hospitalization Among Patients With Atrial Fibrillation and Subtypes of Heart Failure
Source: J Am Heart Assoc. 2026 Jan 14;15(2):e045970. doi: 10.1161/JAHA.125.045970 (PMC12919494; doi:10.1161/JAHA.125.045970)
Supplement: Supplementary file 1 — Table S1 [file JAH3-15-e045970-s001.pdf]

SUPPLEMENTAL MATERIAL

Table S1. Relative median biomarker difference between HFpEF and HFrEF.

Protein	Median HFpEF	Median HFrEF	Relative median HFpEF	Relative median HFrEF	p-value	p-value adjusted
**NT-proBNP (log2)***	**9,53527538**	**10,0505249**	**1**	**1,42924131**	**2,5E-12**	**6,8E-10**
**cTnT-hs (log2)***	**3,47248777**	**3,82272114**	**1**	**1,27476682**	**1,0E-09**	**2,7E-07**
**Renin***	**7,51158293**	**7,87313518**	**1**	**1,28480752**	**1,6E-07**	**4,2E-05**
**BNP***	**4,10283075**	**4,56128965**	**1**	**1,37407323**	**2,4E-07**	**6,8E-05**
**GDF-15 (log2)***	**10,4199602**	**10,6247943**	**1**	**1,15255382**	**5,7E-06**	**0,0015**
**SCF***	**9,67360325**	**9,56611021**	**1**	**0,92819958**	**1,2E-05**	**0,0032**
**ACE2***	**3,92957549**	**4,12990073**	**1**	**1,14895734**	**4,1E-05**	**0,01078**
**IL-6 (log2)***	**1,26303441**	**1,5360529**	**1**	**1,20833333**	**9,0E-05**	**0,024**
**Leptin***	**6,84624058**	**6,57601232**	**1**	**0,82918835**	**0,00012**	**0,031**
DLK-1	5,44430839	5,30767601	1	0,90964001	0,00024	0,063
Cystatin C (log2)	0	0,11103131	1	1,08	0,00026	0,068
IL1RL2	4,62783706	4,5282462	1	0,93329763	0,00028	0,072
MMP-3	7,49287122	7,62138075	1	1,09316375	0,00041	0,10
MMP-9	4,18125557	4,33806294	1	1,11481736	0,00045	0,12
CD5	3,5	3,42	1	0,94605765	0,00046	0,12
hGDNF	2,25	2,32	1	1,04971668	0,00059	0,15
TGF-alpha	1,2	1,25	1	1,03526492	0,0027	0,68
PD-L1	5,02	5,1	1	1,05701804	0,0030	0,76
OSM	2,63	2,74	1	1,07922824	0,0032	0,79
CNTN1	3,20390728	3,15063965	1	0,96375101	0,0032	0,79
AGRP	3,27146548	3,34281319	1	1,05069775	0,0032	0,80
PSP-D	2,75420006	2,84003047	1	1,06129844	0,0071	1
OPN	5,42302791	5,5169028	1	1,06723278	0,0072	1
TM	8,47090417	8,42357072	1	0,96772333	0,0087	1
PIgR	6,44697434	6,47685626	1	1,02092856	0,0087	1
CCL15	7,34480286	7,4406512	1	1,06869364	0,0095	1
TRAIL	7,67	7,62	1	0,96593633	0,0099	1
VEGF-D	7,49270006	7,53415072	1	1,02914814	0,013	1
IL-10RB	6,75	6,71	1	0,97265495	0,013	1
IL-1RT2	5,22884545	5,27125177	1	1,02983008	0,013	1
LOX-1	6,84531743	6,93584593	1	1,06476016	0,015	1
CEACAM8	4,25081043	4,35242407	1	1,0729729	0,020	1
NT-3	1,99	2,04	1	1,03526492	0,021	1
MEPE	3,39742227	3,43624098	1	1,02727234	0,022	1
CXCL10	10,28	10,43	1	1,10956947	0,023	1
SERPINA12	3,74427265	3,60230329	1	0,90628119	0,028	1
U-PAR	4,8253219	4,9024079	1	1,0548852	0,026	1
CCL3	2,84886846	2,90974996	1	1,04310291	0,028	1
FGF-23	4,31908205	4,43750827	1	1,08555003	0,031	1
HO-1	11,6030672	11,6627118	1	1,04220903	0,033	1

PRSS8	9,22088127	9,27685139	1	1,03955791	0,035	1
ADM	7,5674701	7,61655562	1	1,0346089	0,035	1
CD6	3,98	3,94	1	0,97265495	0,038	1
IL-33	1,73	1,73	1	1	0,038	1
CD244	6,02	6	1	0,9862327	0,039	1
TRANCE	4,7	4,62	1	0,94605765	0,041	1
TR	5,29002969	5,22813676	1	0,95800632	0,043	1
CCL16	6,17455052	6,26958735	1	1,06809267	0,043	1
TRAIL-R2	5,84532192	5,895833	1	1,03563173	0,043	1
TLT-2	4,248779	4,17389039	1	0,94941542	0,044	1
HSP 27	10,2619496	10,1922665	1	0,95284727	0,046	1
IL-20	0,79	0,79	1	1	0,047	1
FAS	5,0667276	5,05595524	1	0,99256098	0,047	1

Biomarkers with significant difference between HFpEF and HFrEF according to Wilcoxon−Mann−Whitney test included. * indicates biomarkers with significant difference between HFpEF and HFrEF after multiplicity adjustment.
